# The *goya* mouse mutant reveals distinct newly identified roles for MAP3K1 in the development and survival of cochlear sensory hair cells

**DOI:** 10.1242/dmm.023176

**Published:** 2015-12-01

**Authors:** Andrew Parker, Sally H. Cross, Ian J. Jackson, Rachel Hardisty-Hughes, Susan Morse, George Nicholson, Emma Coghill, Michael R. Bowl, Steve D. M. Brown

**Affiliations:** 1 MRC Mammalian Genetics Unit, MRC Harwell, Oxford, OX11 0RD, UK; 2 MRC Human Genetics Unit, MRC IGMM, University of Edinburgh, Edinburgh, EH4 2XU, UK; 3 The Roslin Institute, University of Edinburgh, Easter Bush, EH25 9RG, UK; 4 Department of Statistics, University of Oxford, Oxford, OX1 3TG, UK

**Keywords:** MAP3K1, Supernumerary outer hair cells, Cochlear development, Sensory hair cell survival, Hearing loss

## Abstract

Mitogen-activated protein kinase, MAP3K1, plays an important role in a number of cellular processes, including epithelial migration during eye organogenesis. In addition, studies in keratinocytes indicate that MAP3K1 signalling through JNK is important for actin stress fibre formation and cell migration. However, MAP3K1 can also act independently of JNK in the regulation of cell proliferation and apoptosis. We have identified a mouse mutant, *goya*, which exhibits the eyes-open-at-birth and microphthalmia phenotypes. In addition, these mice also have hearing loss. The *goya* mice carry a splice site mutation in the *Map3k1* gene. We show that *goya* and kinase-deficient *Map3k1* homozygotes initially develop supernumerary cochlear outer hair cells (OHCs) that subsequently degenerate, and a progressive profound hearing loss is observed by 9 weeks of age. Heterozygote mice also develop supernumerary OHCs, but no cellular degeneration or hearing loss is observed. MAP3K1 is expressed in a number of inner-ear cell types, including outer and inner hair cells, stria vascularis and spiral ganglion. Investigation of targets downstream of MAP3K1 identified an increase in p38 phosphorylation (Thr180/Tyr182) in multiple cochlear tissues. We also show that the extra OHCs do not arise from aberrant control of proliferation via p27KIP1. The identification of the *goya* mutant reveals a signalling molecule involved with hair-cell development and survival. Mammalian hair cells do not have the ability to regenerate after damage, which can lead to irreversible sensorineural hearing loss. Given the observed *goya* phenotype, and the many diverse cellular processes that MAP3K1 is known to act upon, further investigation of this model might help to elaborate upon the mechanisms underlying sensory hair cell specification, and pathways important for their survival. In addition, MAP3K1 is revealed as a new candidate gene for human sensorineural hearing loss.

## Abstract (precis)

**Summary:** The ENU-derived mouse mutant *goya*, reveals, for the first time, multiple roles of MAP3K1 in cochlear development and correct auditory function.

## INTRODUCTION

The signalling pathways underlying epithelial sheet movements are well studied, and have identified the mitogen-activated protein kinase (MAPK) MAP3K1 as having an important role in this process (Takatori et al., 2008; Xia and Kao, 2004; Zhang et al., 2005, 2003). In mice, loss-of-function mutations in the *Map3k1* gene lead to defects in epithelial migration that manifest as an eyes-open-at-birth (EOB) phenotype (Xia and Kao, 2004; Zhang et al., 2003), due to defects in actin polymerisation and c-JUN phosphorylation. Studies in keratinocytes demonstrate that activation of c-Jun N-terminal kinase (JNK) by TGF-β and activin requires MAP3K1, leading to c-JUN phosphorylation, actin-stress-fibre formation and cell migration (Zhang et al., 2005, 2003). Although it is clear that a MAP3K1-JNK cascade is crucial for epithelial-sheet movements during eye organogenesis, it might also be expected to have a role in the development of other epithelia. Indeed, MAP3K1 is required during wound healing, where injury upregulates MAP3K1 and leads to changes in the expression of genes associated with extracellular matrix homeostasis. Conversely, knockdown of MAP3K1 impairs wound healing (Deng et al., 2006). MAP3K1 has also been shown to act independently of JNK during the regulation of cell proliferation and apoptosis in the retina (Mongan et al., 2011).

In humans, *MAP3K1* mutations have been shown to cause 46,XY disorders of sexual development (DSD) (Loke et al., 2014; Pearlman et al., 2010). A number of these mutations have been studied, and they all result in the increased phosphorylation of the downstream MAPK proteins p38 MAPK and ERK1/2.

As part of an *N*-ethyl-*N*-nitrosourea (ENU)-mutagenesis recessive screen, we have identified a mouse mutant, *goya*, which carries a mutation in the *Map3k1* gene. The mutant was identified by its EOB phenotype, and also by the reduction or absence of a response to a click-box test, indicating hearing loss. Homozygous *goya* mice initially develop supernumerary outer hair cells (OHCs) in the inner ear; widespread OHC degeneration is observed by 4 weeks of age, and the mice are profoundly deaf by 9 weeks of age. This identifies a previously unknown role for MAP3K1 in auditory hair cell development and survival. Characterisation of the *goya* mutant provides an opportunity to elaborate upon the requirement of this MAPK in cochlear organogenesis and maintenance.

**TRANSLATIONAL IMPACT**

**Clinical issue**

It is estimated that over 250 million people worldwide experience hearing loss, which occurs commonly in aged individuals and, to a lesser extent, in genetically predisposed children. Hearing loss, which can arise from damage to inner-ear sensory hair cells, is a debilitating condition, particularly when it occurs early in life. At present, hearing loss is irreversible; thus, there is currently much interest in the potential of using regeneration-based therapies to restore hearing function. These strategies aim to either manipulate any remaining hair cells to enable them to re-enter the cycle cell and proliferate, or to induce trans-differentiation of supporting cells into hair cells. Over the last three decades, screening and characterisation of mouse mutants has helped reveal many genes and pathways required for hearing and cell specification in the cochlea. Indeed, the benefit of genetic standardisation and the advantage of being able to image the murine inner ear make the mouse an excellent model organism for auditory research.

**Results**

In this study, the authors describe the *goya* mouse model, which carries an ENU-induced mutation in the *Map3k1* gene. They report that, in addition to a previously reported eyes-open-at-birth phenotype, homozygous *goya* (and homozygous *Map3k1*-null) mice also have an auditory phenotype. At 2 weeks of age, homozygote *goya* mice have supernumerary outer hair cells, and have hearing thresholds similar to wild-type animals. By 9 weeks of age, however, these sensory cells have degenerated and the mice exhibit a severe hearing loss. Interestingly, heterozygous *goya* mice also develop supernumerary outer hair cells, but they do not progressively degenerate nor do these mice develop hearing loss. Supernumerary hair cells can result from aberrant proliferation of progenitor cells in the developing cochlea. However, no increase in the number of proliferating (EdU-positive) cells was observed, and the p27KIP1-defined zone of non-proliferation appeared correctly established, in the embryonic cochleae of mutant mice. Studies into the expression level and phosphorylation status of known MAP3K1 target proteins showed no significant differences. However, an increase in p38 phosphorylation was observed in P1 *goya* inner ears when compared with wild-type controls.

**Implications and future directions**

This work reveals a previously undescribed role for MAP3K1 as a key regulator of cochlear development and hair-cell survival. Interestingly, MAP3K1 is known to regulate many of the genes and proteins currently being investigated for their efficacy in regenerative therapy. However, it is apparent that the exact mechanism by which MAP3K1 performs these distinct roles is highly complex, and will require further investigation. The *goya* model has the potential to provide further insight into the MAP3K1 mechanism in the context of hearing development, and could also unveil other genes and pathways required for hearing. Moreover, the model could be used as a platform to test the efficacy of candidate regenerative therapies.

## RESULTS

### Identification of a mouse mutant with eye, vision and auditory defects

The *goya* mouse mutant was identified, from a recessive ENU-mutagenesis phenotype-driven screen, as having EOB (see Fig. S1A). In the adult EOB mice, eye pathology is highly variable, ranging from microphthalmic, to apparently normal, to bulging (see Fig. S1B). However, all EOB mice failed to respond in an optokinetic drum visual-function assay (data not shown). Rosetting was observed in the retinal layers, although, at postnatal day 0 (P0), the structure of tight junctions and the outline of retinal pigment epithelium (RPE) cells appeared normal (see Fig. S1C). Additionally, these mice also had a reduced Preyer's response to a click-box auditory stimulus. Given the interesting combination of eye and auditory defects, we proceeded to identify the *goya* mutation and further explore the deafness phenotype.

### *goya* is caused by a point mutation in the *Map3k1* gene

Using single-nucleotide polymorphism (SNP)-based mapping, the *goya* phenotype was localized to a 24.7 Mb region on chromosome 13, between SNPs rs13481942 and rs6316705. Within the interval there was a strong candidate, *Map3k1*, with mice deficient for this gene having previously been shown to display EOB (Juriloff et al., 2004; Yujiri et al., 2000; Zhang et al., 2003). There have been no reports of hearing loss in *Map3k1* mouse models. *Map3k1* is highly expressed in the migrating leading edge of the eyelid epithelium and it is thought that, in its absence, the migration of these cells is impaired, leading to the failure of eyelid closure during embryogenesis (Xia and Kao, 2004; Zhang et al., 2005, 2003). Investigation of a kinase-deficient *Map3k1^tm1Yxia/tm1Yxia^* allele showed that retinal phenotypes, including increased proliferation and subsequent apoptosis of Müller glial cells, were due to a pathway that is separate to eyelid closure, highlighting the multiple roles of MAP3K1 in cellular development and survival (Mongan et al., 2011).

**Fig. 1.**
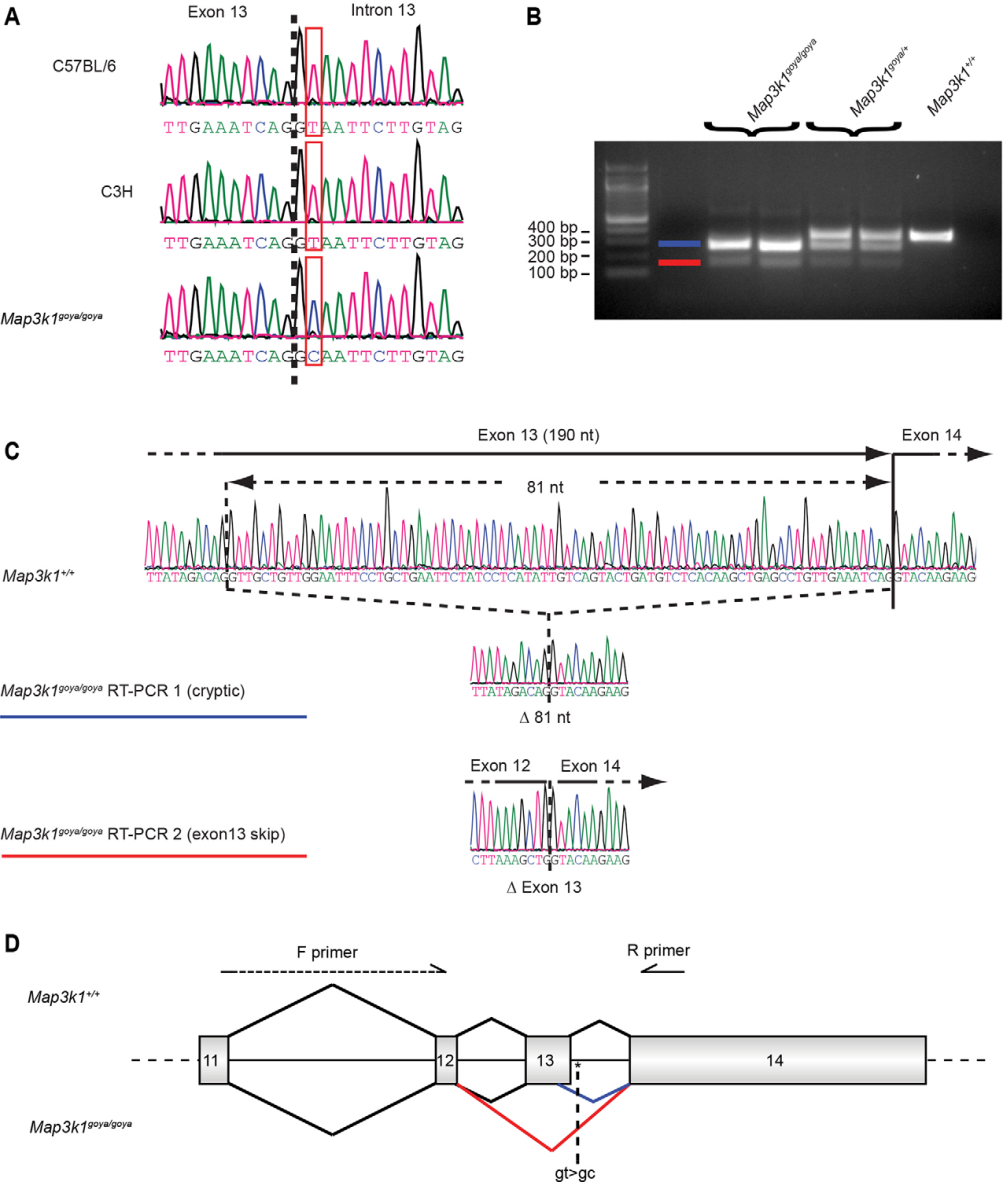
***Map3k1* is mutated in *goya* mice.** (A) Genomic DNA sequence traces showing the *Map3k1* exon 13 donor splice site in the parental strains (C57BL/6 and C3H), and homozygous mutant (*Map3k1^goya/goya^*) mice. The affected nucleotide is boxed (T in parental strains and C in *Map3k1^goya/goya^*) and the exon/intron border is indicated by the dashed line. (B) RT-PCR analysis of RNA extracted from the organ of Corti. For *Map3k1^+/+^*, a single 341-bp amplicon corresponding to the expected wild-type *Map3k1* sequence was observed. For *Map3k1^goya/goya^*, no wild-type amplicon was observed; instead, two smaller amplicons were identified (denoted by blue and red lines). All three amplicons were found for *Map3k1^goya/+^*. (C) Sanger sequencing reveals aberrant splicing in *Map3k1^goya/goya^*. (Top) Sequencing of the single *Map3k1^+/+^* product confirms normal splicing of exons 12/13/14; (middle) sequencing of the larger *Map3k1^goya/goya^* product (blue) reveals the use of a cryptic splice site within exon 13, resulting in an in-frame deletion of 81 nucleotides; (bottom) sequencing of the smaller *Map3k1^goya/goya^* product (red) reveals exon 12 splicing directly to exon 14, with complete skipping of exon 13. (D) Cartoon depicting the aberrant splicing events occurring in *Map3k1^goya/goya^* mice. Exons are depicted as numbered boxes, and the ‘cryptic’ and ‘exon 13 skip’ splicing events are shown as blue and red lines, respectively. The location of the *goya* mutation is shown.

Sanger sequencing of the *Map3k1* gene exons and intron/exon boundaries revealed a single-nucleotide change in the intron 13 splice donor site (IVS13+2T>C) of affected mice (Fig. 1A). To ascertain the effect of the *goya* mutation on splicing, we performed reverse-transcriptase PCR (RT-PCR) analysis of RNA isolated from postnatal day 1 (P1) organ of Corti dissected from *goya* littermate mice. For *Map3k1^+/+^*, the expected product of 341 bp was obtained (Fig. 1B). However, for *Map3k1^goya/goya^*, the wild-type product was absent and two smaller products could be seen (Fig. 1B). Sanger sequencing of the *Map3k1^+/+^* RT-PCR product showed that exons 12, 13 and 14 were correctly spliced, whereas sequencing of the two *Map3k1^goya/goya^* products showed aberrant splicing (Fig. 1C). The more abundant mutant product (*Map3k1^goya/goya^* RT-PCR 1) demonstrates the use of a cryptic splice donor site within exon 13 (Fig. 1C). In this case, 81 nucleotides are skipped, leaving the transcript in-frame but producing a protein with an internal deletion of 27 amino acids. The less abundant mutant product (*Map3k1^goya/goya^* RT-PCR 2) showed complete skipping of exon 13 (190 nucleotides), with exon 12 spliced directly to exon 14 (Fig. 1C). Translation of this transcript would lead to the production of a protein containing the first 723 amino acids of MAP3K1 followed by a frame-shift and the incorporation of seven novel amino acids before a stop codon is encountered. If produced, this truncated protein would lack the C-terminal 770 amino acids of MAP3K1, including the kinase domain.

### The *goya* mutant is severely deaf

**Fig. 2.**
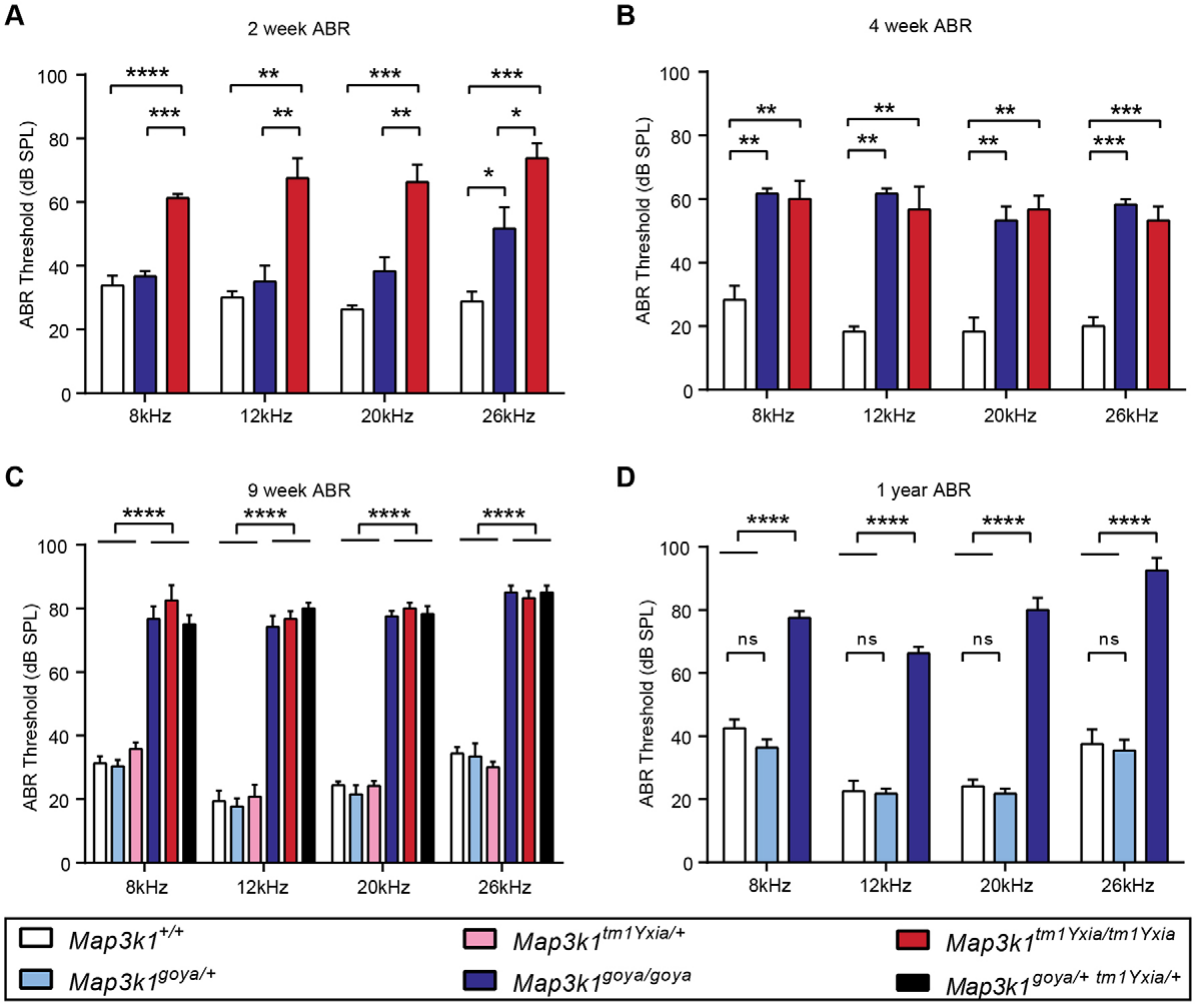
***Map3k1*-deficient mice have progressive hearing loss.** (A) Average ABR thresholds of *Map3k1^+/+^* (*n*=4), *Map3k1^goya/goya^* (*n*=3) and *Map3k1^tm1Yxia/tm1Yxia^* (*n*=4) mice at 2 weeks of age (P16). *Map3k1^tm1Yxia/tm1Yxia^* show significantly elevated thresholds at all frequencies when compared to wild-type or *Map3k1^goya/goya^* mice. *Map3k1^goya/goya^* mice only show a significant difference from wild-type at 26 kHz. (B) At 4 weeks of age, *Map3k1^goya/goya^* (*n*=3) and *Map3k1^tm1Yxia/tm1Yxia^* (*n*=3) mice have significantly elevated average ABR thresholds (+30-40 dB) when compared to *Map3k1^+/+^* (*n*=3) mice across all frequencies tested. (C) By 9 weeks of age, *Map3k1^goya/goya^* (*n*=6), *Map3k1^tm1Yxia/tm1Yxia^* (*n*=6) and *Map3k1^goya/tm1Yxia^* (*n*=6) mice exhibit severe hearing loss, as demonstrated by ABR thresholds of 50-60 dB above *Map3k1^+/+^* mice. Mice heterozygous for both the *goya* (*n*=13) and *tm1Yxia* (*n*=6) alleles have thresholds not significantly different from wild type at 9 weeks of age, showing that they do not suffer the same progressive hearing loss as homozygote mice. (D) ABR performed at 1 year of age showed that *Map3k1^goya/+^* (*n*=6) mice have similar thresholds compared to *Map3k1^+/+^* (*n*=5) mice. Also, *Map3k1^goya/goya^* (*n*=7) mice have thresholds similar to those measured at 9 weeks of age, suggesting no further decline in hearing function. Data shown are mean±s.e.m.; *P*-values calculated using one-way ANOVA with TUKEY post-hoc analysis: *P≤0.05, **P≤0.01, ***P≤0.001, ****P≤0.0001, ns, not significant.

To confirm *Map3k1* as the causative gene, we mated mice carrying the *goya* mutation to mice carrying the *Map3k1^tm1Yxia^* allele. We performed auditory brainstem response (ABR) analysis on both *Map3k1^goya^* and *Map3k1^tm1Yxia^* heterozygote and homozygote mice as well as compound heterozygote and wild-type mice. At 9 weeks of age, *Map3k1^goya/+^* and *Map3k1^tm1Yxia/+^* heterozygous mice had normal auditory thresholds, similar to wild-type (*Map3k1^+/+^*) mice, whereas both *Map3k1^goya/goya^* and *Map3k1^tm1Yxia/tm1Yxia^* homozygotes showed a significant hearing loss with average ABR thresholds of ∼80 decibels sound pressure level (dB SPL) across all frequencies tested (*P*≤0.0001) (Fig. 2C). Importantly, the compound heterozygous mice (*Map3k1^goya^*^/*tm1Yxia*^) also have elevated ABR thresholds, similar to those of homozygote mice. Failure of these models to complement confirms that the ENU-induced *Map3k1* mutation underlies the *goya* phenotype. To investigate the onset of hearing loss, we performed ABR testing of homozygote and wild-type mice at 2 and 4 weeks of age. At 2 weeks of age, *Map3k1^goya/goya^* mice had ABR thresholds similar to those of wild-type mice at all but the highest frequency tested (26 kHz), whereas *Map3k1^tm1Yxia/tm1Yxia^* mice showed elevated thresholds at all frequencies tested (Fig. 2A). At 4 weeks of age, both homozygous mutants exhibited significantly elevated ABR thresholds (+30-40 dB) at all frequencies tested when compared to wild-type controls (Fig. 2B).

To determine the longitudinal effects of the *goya* mutation on hearing function, we performed ABR on mice at 1 year of age. *Map3k1^goya/+^* mice had ABR thresholds similar to those of wild-type mice, demonstrating that *goya* heterozygotes do not develop late-onset hearing loss (Fig. 2D). Moreover, there is no further decline in auditory function of *Map3k1^goya/goya^* animals between 9 weeks and 1 year (Fig. 2C,D). To investigate vestibular effects of the mutation, we performed swim tests on 1-year-old *Map3k1^goya/goya^* mice, and no overt vestibular dysfunction was observed.

### Hair-cell abnormalities in the *Map3k1* mutants

**Fig. 3.**
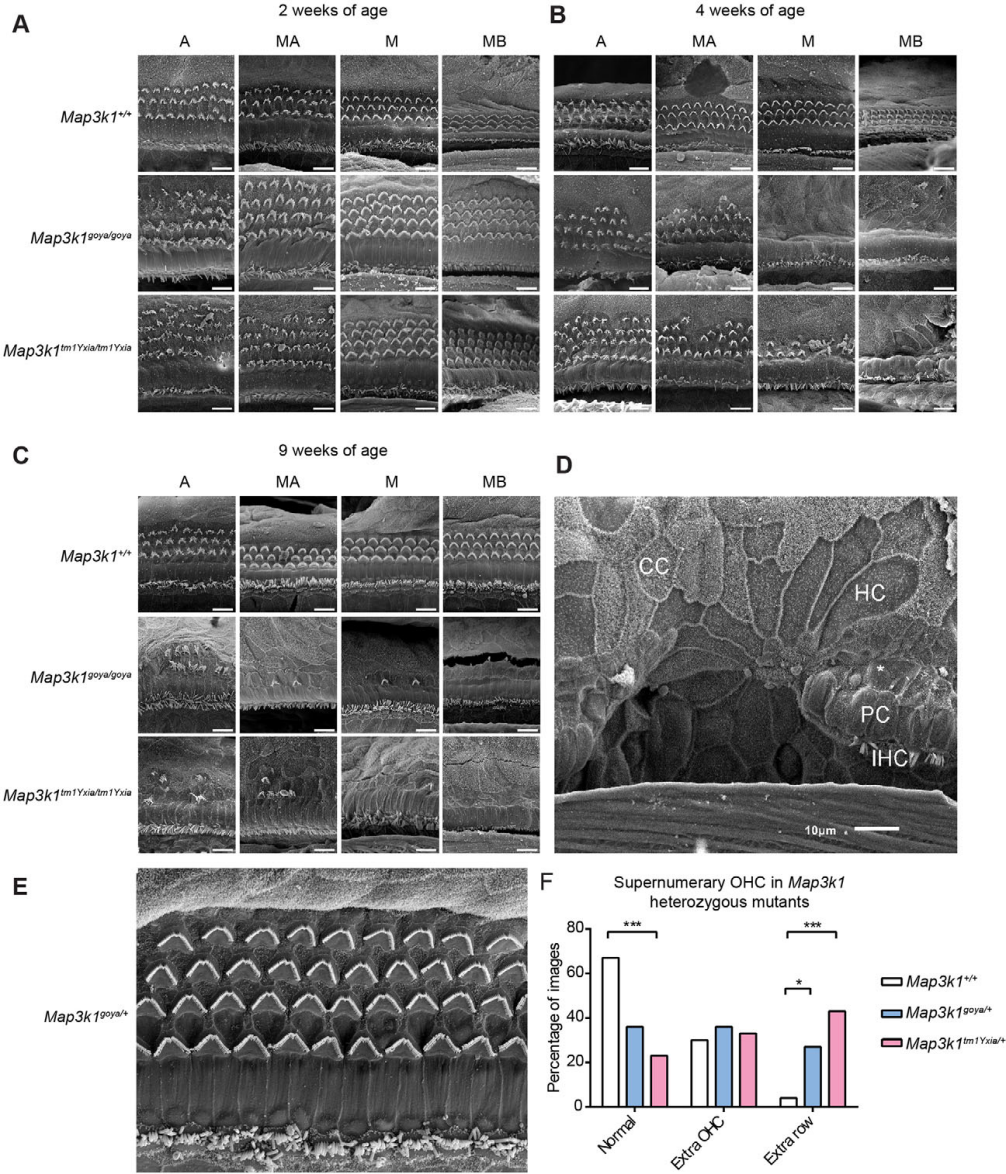
**Mice lacking functional MAP3K1 develop supernumerary OHCs and show progressive degeneration of OHCs.** (A-C) Representative scanning electron micrographs from the apical (‘A’), mid-apical (MA), mid (M) and mid-basal (MB) turns of the organ of Corti from *Map3k1^+/+^*, *Map3k1^goya/goya^* and *Map3k1^tm1Yxia/tm1Y^^xia^* mice at 2, 4 and 9 weeks of age. Both *Map3k1^goya/goya^* and *Map3k1^tm1Yxia/tm1Yxia^* mice have an extra row of OHCs at 2 weeks of age. A progressive loss of OHCs is seen between 2 and 9 weeks of age in both homozygous mutants, but not in wild-type cochleae. An apical-to-basal increase in severity of degeneration was also observed. (D) Scanning electron micrograph demonstrating the rosette-like cellular formation in a 9-week-old *Map3k1^tm1Yxia/tm1Yxia^* mouse. The remains of some IHC stereocilia bundles can be seen (IHC), all OHC are missing (*) and pillar cells (PC) have been replaced in the rosette formation with Hensen (HC)-like and Claudius (CC)-like cells (all scale bars represent 10 µm). (E) Scanning electron micrograph showing an extra row of OHCs in the mid-basal region of a 9-week-old *Map3k1^goya/+^* cochlea. (F) Clustered histogram representing the percentage of total images captured from *Map3k1^+/+^* (*n*=54), *Map3k1^goya/+^* (*n*=11) and *Map3k1^tm1Yxia/+^* (*n*=30) mice containing three rows of OHCs (normal), extra OHCs (1-4 OHCs in addition to the three normal rows), or extra rows of OHCs (≥5 OHCs in a continuous line, in addition to the three normal rows). Images of cochleae from both heterozygote alleles and *Map3k1^+/+^* mice showed isolated extra OHCs; however, 27% of *Map3k1^goya/+^* and 43% of *Map3k1^tm1Yxia/+^* heterozygote images contained extra rows of OHCs, significantly higher than the 4% of *Map3k1^+/+^* images. The percentage of *Map3k1^tm1Yxia/+^* images containing the normal three rows of OHCs was also significantly lower when compared to *Map3k1^+/+^* (*P<0.05, ***P<0.0001, Fisher's exact test, see Materials and Methods and Table S3 for estimates confidence intervals and *P*-values).

Given the elevated auditory thresholds of *Map3k1^goya/goya^* and *Map3k1^tm1Yxia/tm1Yxia^* mice, we proceeded to examine in detail the ears of these mice. Hematoxylin and eosin (H&E) staining of cochlear sections suggested cellular degeneration within the organ of Corti of 9-week-old homozygote mice; however, other inner-ear structures such as Reissner's membrane, stria vascularis and spiral ganglion neurons (SGNs) appeared normal (data not shown). Additionally, no defects in middle-ear morphology were observed. To further assess the organ of Corti, we used scanning electron microscopy (SEM) to examine the ultrastructure of the sensory epithelium. At 2 weeks of age, *Map3k1^goya/goya^* and *Map3k1^tm1Yxia/tm1Yxia^* mice showed a normal cellular arrangement of the sensory epithelium with the exception that both mutants had more OHCs than did wild type (Fig. 3A). The additional OHCs were organised as an extra row that extended largely throughout the cochlear regions examined (Fig. 3A). However, by 4 weeks of age both mutants showed degeneration of OHCs with an increasing severity from apex-to-base (Fig. 3B). By 9 weeks of age both mutants showed further degeneration. Conversely, no OHC loss was observed in wild-type mice by 9 weeks of age (Fig. 3C). At 2, 4 and 9 weeks of age, inner hair cell (IHC) morphology appeared normal in all except *Map3k1^tm1Yxia/tm1Yxia^* cochlea at 9 weeks, which showed a slight reduction in number again with an apical-to-basal gradient (Fig. 3C). Although at 9 weeks of age the majority of IHCs were unaffected, the extent of degeneration in small patches of the *Map3k1^tm1Yxia/tm1Yxia^* organ of Corti was very severe. IHCs, OHCs and pillar cells had disappeared and rosette-like formations of what seemed to be Claudius and Hensen cells had formed in their place (Fig. 3D). A similar pattern of cellular remodelling has been previously noted, in post-aminoglycoside-damaged cochlea (Taylor et al., 2012). Interestingly, similar to homozygous mutants, *Map3k1^goya/+^* and *Map3k1^tm1Yxia/^*^+^ mice also displayed extra OHCs compared to wild-type mice, but, unlike homozygous mutants, no OHC degeneration was observed at any time point (Fig. 3E). Further investigation shows that, similar to wild type, the heterozygote mutants have episodic patches of extra OHCs. However, unlike wild type, the heterozygote mutants show an increase in the occurrence of extra rows of OHCs, which are present in 43%, 27% and 4% of images from *Map3k1^tm1Yxia/^*^+^, *Map3k1^goya/+^* and wild-type mice, respectively (Fig. 3F). These data suggest roles for MAP3K1 in sensory-hair-cell development and survival.

**Fig. 4.**
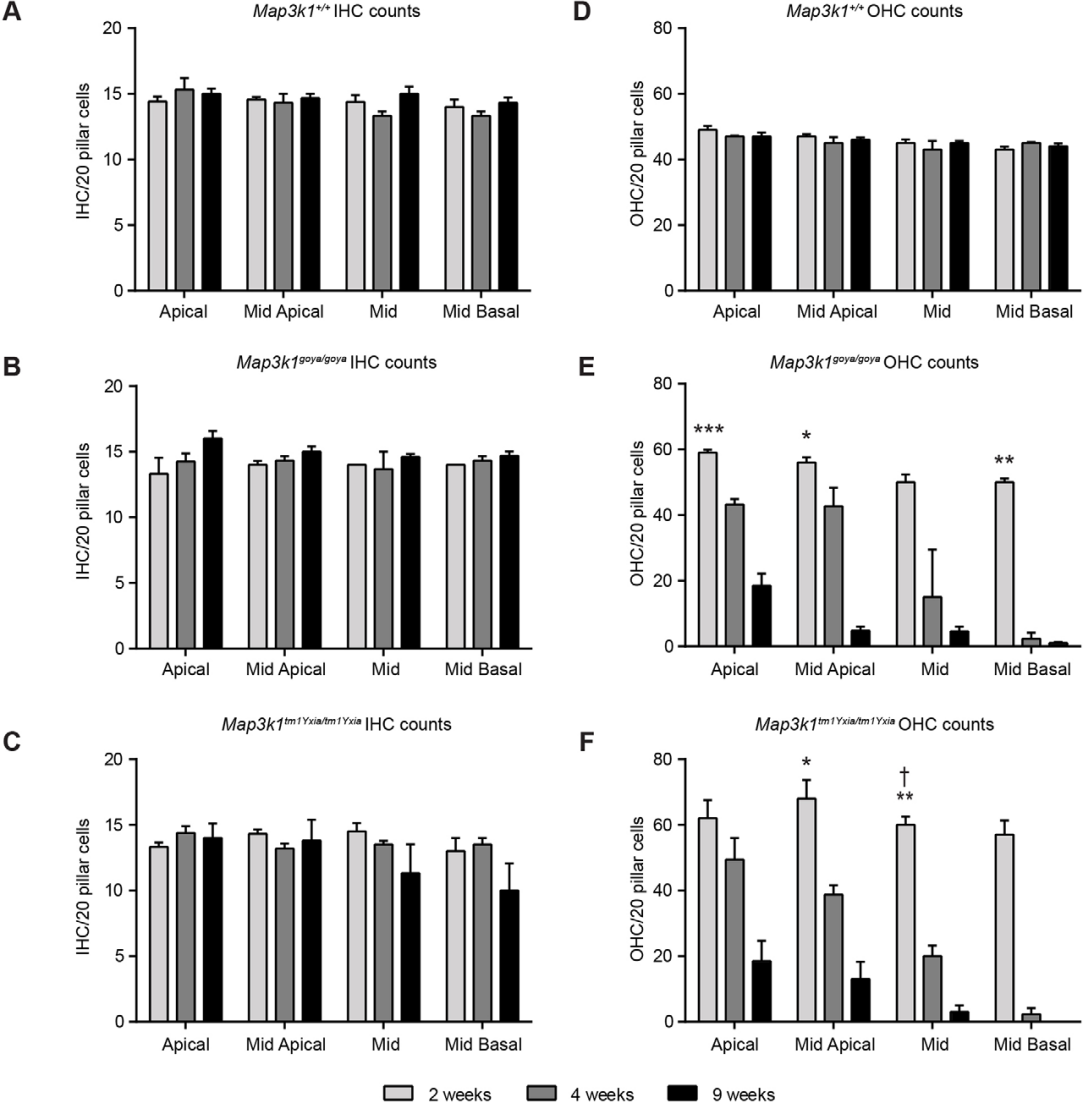
**Quantification of hair-cell loss in the organ of Corti of *Map3k1^goya/goya^* and *Map3k1^tm1Yxia/tm1Yxia^* mice.** (A-C) Average number of IHCs in contact with 20 pillar cells at 2, 4 and 9 weeks of age. No significant differences in IHC number were observed in wild-type (+/+; A), *Map3k1^goya/goya^* (B) and *Map3k1^tm1Yxia/tm1Yxia^* (C) mice, although, by 9 weeks of age, reduced numbers of IHCs were observed in some of the *Map3k1^tm1Yxia/tm1Yxia^* cochleae. (D-F) Average number of wild-type (+/+; D), *Map3k1^goya/goya^* (E) and *Map3k1^tm1Yxia/tm1Yxia^* (F) OHCs in contact with 20 pillar cells at 2, 4 and 9 weeks of age. *t*-tests were performed to compare the mean numbers of OHCs between genotypic groups at 2 weeks of age (see Materials and Methods). *Map3k1^goya/goya^* and *Map3k1^tm1Yxia/tm1Yxia^* mice have more OHCs than do wild type. This difference was significant in the apical (***P≤0.001), mid-apical (*P≤0.05) and mid-basal (**P<0.01) turns in *Map3k1^goya/goya^*. In *Map3k1^tm1Yxia/tm1Yxia^* mice, the extra number of OHCs was significantly higher than in wild type in the mid-apical (*P<0.05) and mid (**P<0.01) turns. In the mid turn of *Map3k1^tm1Yxia/tm1Yxia^* cochleae, significantly more OHCs were observed than in *Map3k1^goya/goya^* (^†^*P*<0.05); however, there were no other obvious differences between the mutant alleles. By 4 weeks of age, nearly all *Map3k1^goya/goya^* and *Map3k1^tm1Yxia/tm1Yxia^* OHCs were missing in the mid-basal turn and, in the mid turn, we observed variable levels of OHC loss in *Map3k1^goya/goya^* cochleae, and substantial loss in *Map3k1^tm1Yxia/tm1Yxia^* cochleae. In the mid-apical and apical turns, OHC loss was evident but not to the extent of the mid and mid-basal turns. By 9 weeks of age the majority of OHCs were missing in the mid-basal and mid turns of both *Map3k1^goya/goya^* and *Map3k1^tm1Yxia/tm1Yxia^* cochleae, and severe loss was seen in the mid and mid-apical turns. No significant difference in OHC numbers were seen across the time points in wild-type cochleae. The rate of decrease in hair-cell number over time was also analysed and found to be highly significant in both homozygous mutants (see Materials and Methods and Tables S1 and S2 for *P*-values). Data shown are mean±s.e.m., *n*≥3 for genotype at each cochlear turn: *P≤0.05, **P≤0.01, ***P≤0.001.

To assess the progressive nature of cell loss in the different regions of the cochlear spiral, and to allow comparison between genotypes, sensory-cell counts were performed. At 2 and 4 weeks of age, IHC numbers were similar between *Map3k1^+/+^*, *Map3k1^goya/goya^* and *Map3k1^tm1Yxia/tm1Yxia^* mice. At 9 weeks of age, IHC numbers were similar between *Map3k1^+/+^* and *Map3k1^goya/goya^* mice, but a trend for reduced numbers of IHCs in the basal region of *Map3k1^tm1Yxia/tm1Yxia^* cochleae was observed (Fig. 4A-C). Up to 9 weeks of age, *Map3k1^+/+^* mice showed consistent numbers of OHCs across the different regions of the cochlea (Fig. 4D). At 2 weeks of age, *Map3k1^goya/goya^* and *Map3k1^tm1Yxia/tm1Yxia^* mice had increased numbers of OHCs compared to wild type, which was significant for most of the regions assessed (Fig. 4D-F). At 4 weeks of age, degeneration of OHCs progressed in *Map3k1^goya/goya^* and *Map3k1^tm1Yxia/tm1Yxia^* mice. In the apical, mid-apical, mid and mid-basal regions of the cochlear spiral, *Map3k1^goya/goya^* mutants showed an average reduction in OHC numbers of 26%, 24%, 70% and 95%, respectively, whereas *Map3k1^tm1Yxia/tm1Yxia^* mutants showed reductions of 20%, 43%, 67% and 96%, respectively (Fig. 4E,F). A similar apical-to-basal increase in severity was also observed in 9-week-old mutants (Fig. 4).

To determine statistical significance, the rate of hair-cell loss was estimated under a Poisson generalised linear model (see Materials and Methods). There was no statistical support for IHC loss in any genotypic group (see Tables S1, S2). There was strong statistical support for OHC loss in the *Map3k1^goya/goya^* and *Map3k1^tm1Yxia/tm1Yxia^* genotypic groups (but no evidence in the wild-type group; see Table S2). The rate of OHC loss in the mutant groups increased consistently from apical-to-basal turns (see Table S1). The rate of OHC loss did not differ significantly between the two mutant groups. These statistical analyses quantify and corroborate the obvious effects visible in Fig. 3.

### Localisation of MAP3K1 to the inner ear

**Fig. 5.**
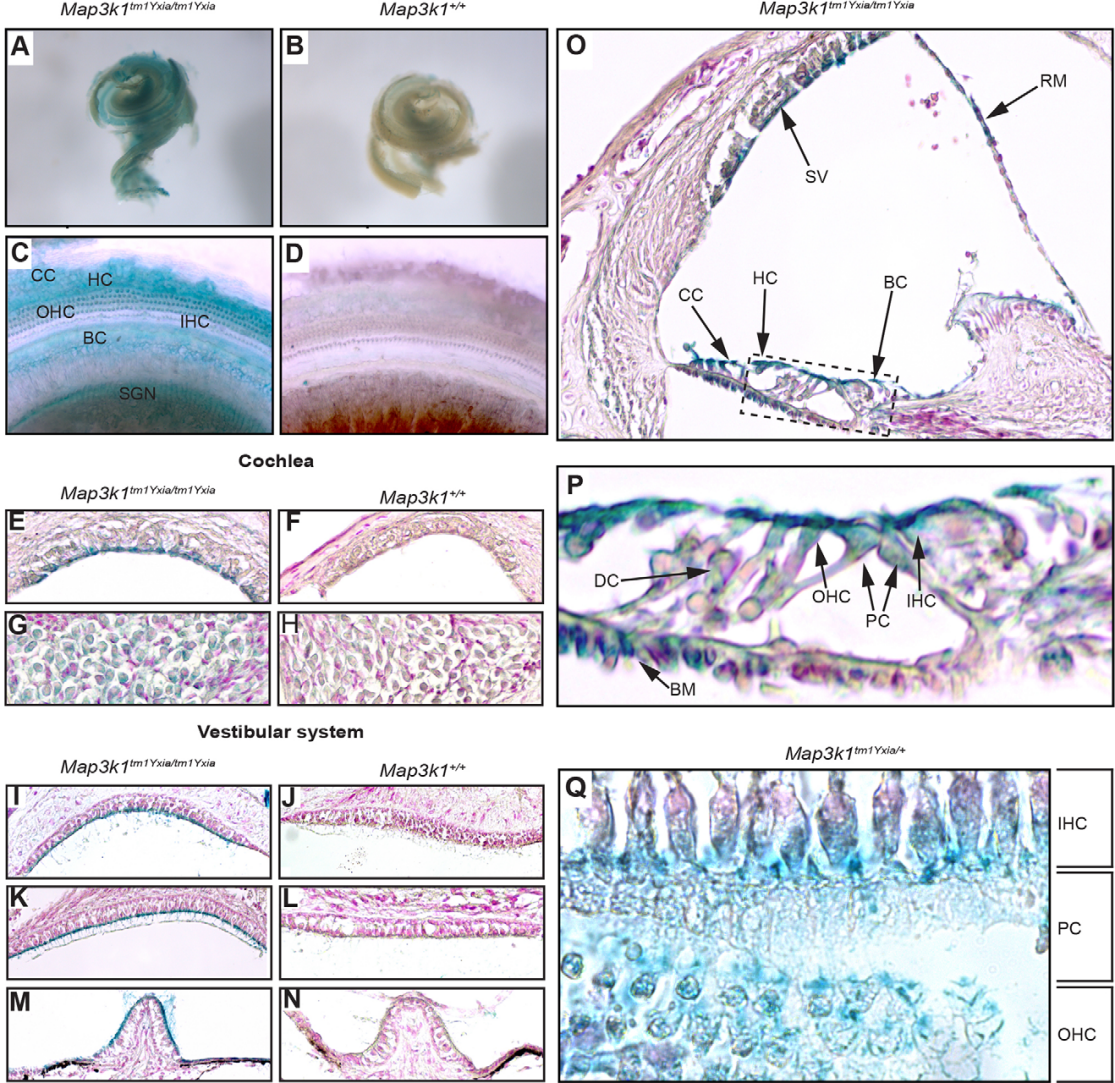
**Map3k1–β-galactosidase expression in the inner ear of P12 *Map3k1^tm1Yxia/tm1Yxia^* and *Map3k1^tm1Yxia/+^* mice.** (A,B) X-Gal-stained cochleae from *Map3k1^tm1Yxia/tm1Yxia^* and *Map3k1^+/+^* mice, respectively. The *Map3k1^tm1Yxia/tm1Yxia^* cochlea (A) shows widespread staining owing to the presence of a MAP3K1–β-galactosidase fusion protein in these mice. (C,D) Extended focus images showing staining in the cochlear duct. In *Map3k1^tm1Yxia/tm1Yxia^* cochlea (C), strong staining can be observed in Claudius cells (CC), Hensen cells (HC), outer hair cells (OHC), inner hair cells (IHC), border cells of the internal spiral sulcus (BC) and spiral ganglion neurons (SGN). This staining is not observed in the *Map3k1^+/+^* cochlea (D). (E-N) X-Gal-stained sagittal sections of the cochleae and vestibular systems from *Map3k1^tm1Yxia/tm1Yxia^* and *Map3k1^+/+^* mice: (E,F) stria vascularis, (G,H) spiral ganglion neurons, (I,J) sacular macula, (K,L) utricular macula, (M,N) crista ampularis. X-Gal-positive staining is present in the marginal cells of the stria vascularis (E), the spiral ganglion neurons (G) and the apical surfaces of supporting cells and hair cells in all of the otolithic organs in the vestibular system of *Map3k1^tm1Yxia/tm1Yxia^* mice. (O) Shows the distribution of MAP3K1–β-galactosidase in the cochlear duct of *Map3k1^tm1Yxia/tm1Yxia^* mice. Staining is observed in the stria vascularis (SV), Reissner's membrane (RM), Claudius cells (CC), Hensen cells (HC) and border cells of the internal spiral sulcus (BC). (P) An enlargement of the organ of Corti (dashed box in panel O), showing MAP3K1–β-galactosidase expression in the apical surface of IHC, OHC, Deiters' cells (DC), pillar cells (PC) and tympanic border cells of the basilar membrane (BM). (Q) Transverse section of the organ of Corti from a *Map3k1^tm1Yxia/+^* mouse, highlighting more clearly X-Gal-positive staining in the IHCs, PCs and OHCs.

Commercially available anti-MAP3K1 antibodies have poor specificity; therefore, we utilized the *Map3k1^tm1Yxia^* allele, which produces a MAP3K1–β-galactosidase fusion protein (Xia et al., 2000; Zhang et al., 2003). Using X-Gal staining, widespread expression of MAP3K1–β-galactosidase was observed in *Map3k1^tm1Yxia/tm1Yxia^* cochleae (Fig. 5A). Closer examination of the cochlear duct showed staining in IHCs and OHCs, border cells of the internal spiral sulcus, Claudius and Hensen cells, as well as SGNs (Fig. 5C). No labelling was observed in wild-type cochleae (Fig. 5B,D). Histological sections confirm the whole-mount localisation data and also reveal expression in cell types such as Deiters' cells, pillar cells, Reissner's membrane, marginal cells in the stria vascularis and the tympanic border cells of the basilar membrane (Fig. 5E,G,O,P). A transverse section of the organ of Corti also highlighted the expression of the fusion protein in IHCs and OHCs (Fig. 5Q). In addition to the expression in the cochlea, staining was observed in the vestibular system, including the apical surface of supporting cells and hair cells in both otolithic organs and the cristae of the semi-circular canals (Fig. 5I,K,M). Positive staining in all *Map3k1^tm1Yxia/+^* samples mirrored that of homozygotes, whereas no staining was seen in either the vestibular system or cochleae of *Map3k1^+/+^* mice (Fig. 5F,H,J,L,N).

### Aberrant proliferation is not the cause of supernumerary OHCs in *Map3k1* mutant mice

**Fig. 6.**
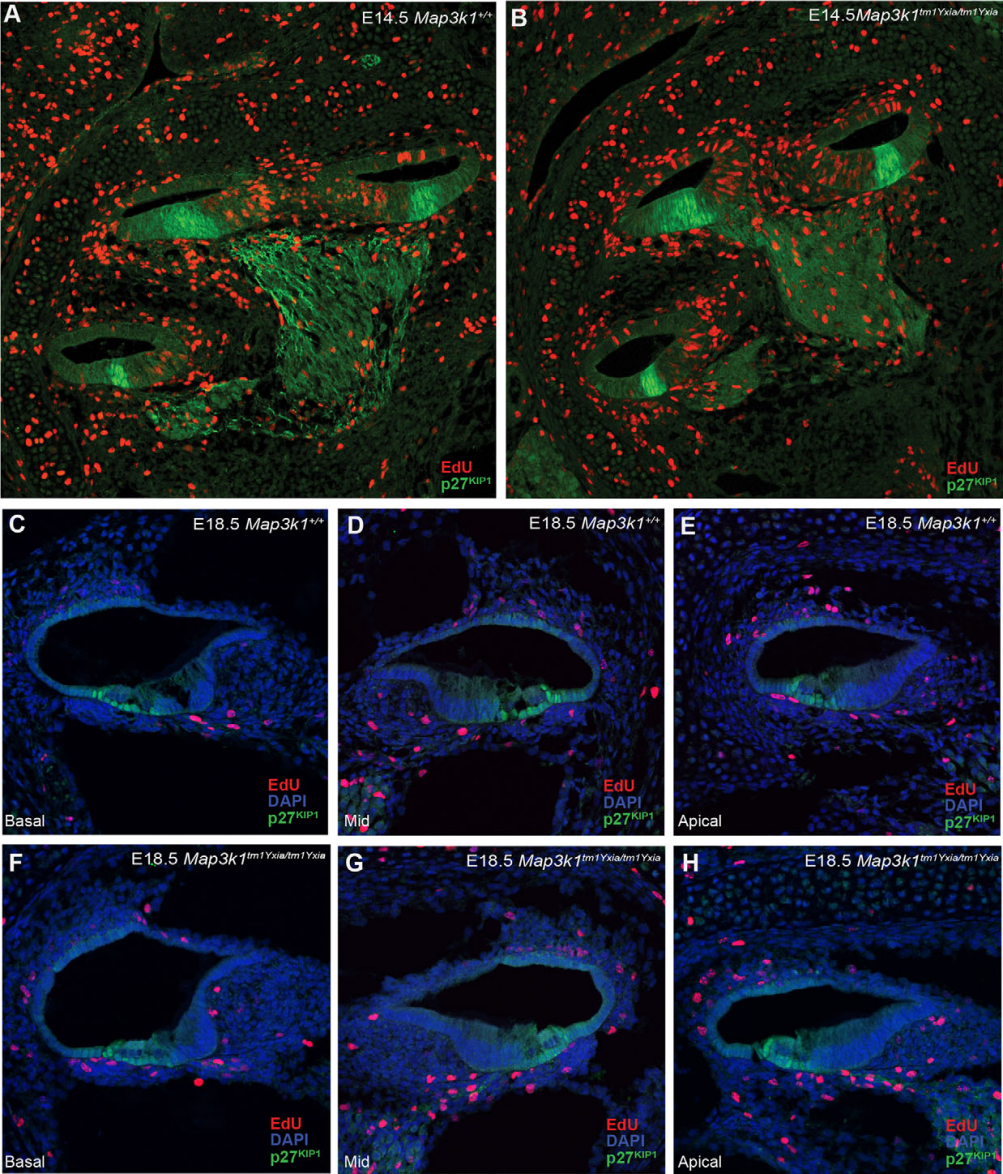
**Analysis of proliferation and p27KIP1 localisation** **in the developing *Map3k1^tm1Yxia/tm1Yxia^* cochlea.** Immunodetection of EdU-positive nuclei (red) and anti-p27KIP1 (green) and DAPI (to highlight nuclei in panels C-H). Pregnant females from timed matings were injected with EdU twice at 2-h intervals before embryos were harvested at E14.5 (A,B) or E18.5 (C-H). Localisation of p27KIP1 is unaffected in E14.5 *Map3k1^tm1Yxia/tm1Yxia^* and *Map3k1^+/+^* cochleae (A,B). The absence of EdU-positive nuclei in the region of p27KIP1 expression indicates that the zone of non-proliferation (ZNP) is established correctly in *Map3k1^tm1Yxia/tm1Yxia^* mutant cochlea. At E18.5, we again saw no difference between the genotypes in p27KIP1 localisation, and found no evidence of proliferating cells, as denoted by EdU-positive nuclei in the cochlear duct epithelia, in any of the cochlear turns (C-H).

The cyclin-dependent kinase inhibitor *p27^Kip1^* is a key regulator that arrests the cell cycle at G1. Its expression in the developing cochlea produces a zone of non-proliferation (ZNP), and these cells subsequently differentiate into sensory hair cells and supporting cells. It is known that these ZNP cells undergo their final division by embryonic day 14.5 (E14.5). However, in *p27^Kip1^* homozygous-null mice, there is an extended period of pro-sensory precursor-cell proliferation leading to increased numbers of hair cells and supporting cells (Lowenheim et al., 1999). We used 5-ethynyl-2′-deoxyuridine (EdU) to investigate whether proliferation was increased or extended in the developing *Map3k1^tm1Yxia/tm1Yxia^* mutant cochlea. In addition, we used an anti-p27KIP1 antibody to investigate for possible regulatory defects associated with a MAP3K1 deficiency. Fig. 6 shows that p27KIP1 localisation is unaffected in the *Map3k1^tm1Yxia/tm1Yxia^* cochlea at E14.5 (Fig. 6A,B), and the lack of EdU-positive nuclei in the region of the cochlea duct that positively immunolabelled for p27KIP1 indicates that the ZNP is established correctly. At E18.5, we again found no difference in localisation of p27KIP1 in mutant cochleae. Moreover, no increase in the number of proliferating cells was seen in the cochlear duct of *Map3k1^tm1Yxia/tm1Yxia^* mice compared to *Map3k1^+/+^* littermate controls (Fig. 6C-H). Together, these data suggest that increased or extended proliferation of pro-sensory precursor cells is not the cause of extra OHCs in *Map3k1* mutant mice.

### The *goya* mutation in *Map3k1* results in an increase of p38 phosphorylation in P1 mouse inner ears

To investigate the effects of the *goya* mutation on MAPK pathway targets, we used both gene expression and immunodetection strategies. We extracted RNA from isolated P1 cochlear ducts and performed qRT-PCR to investigate the expression of *Map3k1* and genes likely to be involved in MAP3K1 signalling [*Ccna1* (cyclin A1), *Ccnd1* (cyclin D1), *Lgr5*, *Dhfr*, *Axin2*, *Ctnbb1* (β-catenin), *E2f1*, *Rb1*] (Loke et al., 2014; Mongan et al., 2011). In addition, expression of JNK targets, *Jun* and *Fos*, were also analysed. Although some differences were observed between genotypes, we did not see any notable fold changes in expression of any of the genes investigated (see Fig. S2A).

**Fig. 7.**
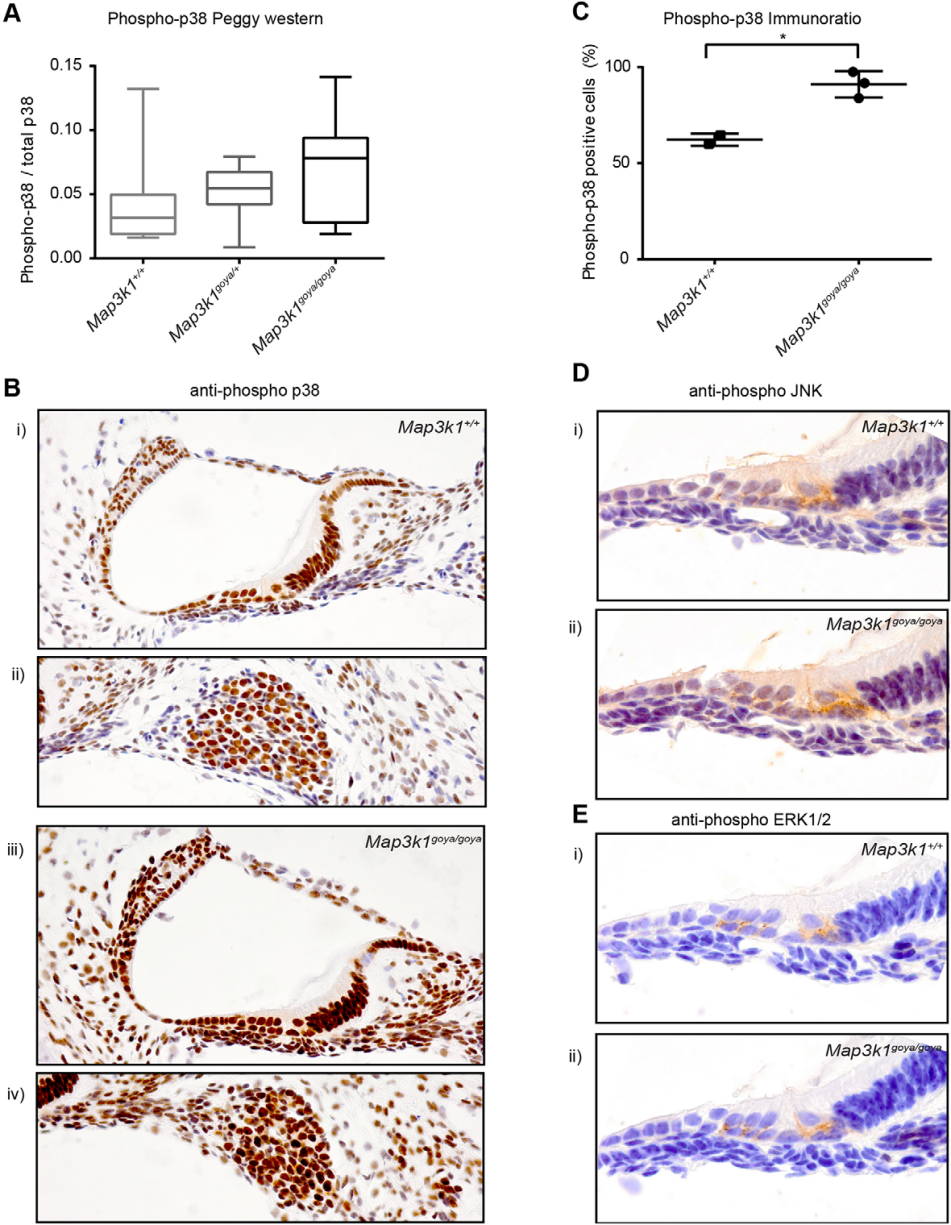
**The inner ears of P1 *Map3k1^goya/goya^* mice exhibit increased levels of p38 phosphorylation.** (A) Box plot of the ratio of phosphorylated p38 to total p38 in *goya* inner-ear total protein lysates from *Map3k1^+/+^*, *Map3k1^goya/+^*and *Map3k1^goya/goya^* mice. Three ears (one each from separate mice) were pooled for each lysate (number of lysates: *Map3k1^+/+^n*=8, *Map3k1^goya/+^ n*=7, *Map3k1^goya/goya^*, *n*=8). A trend of increased p38 phosphorylation is observed in *Map3k1^goya/goya^* inner-ear lysates when compared to *Map3k1^+/+^* (*P*=0.282). Average *Map3k1^goya/+^* p38 phosphorylation levels are intermediate between *Map3k1^+/+^* and *Map3k1^goya/goya^*. (B-E) Immunohistochemical analysis of P1 *Map3k1^goya/goya^* cochlea confirms the increased level of p38 phosphorylation observed in the Peggy Western data. Panels Bi and iii show phosphorylated p38 expression in the mid turn of the cochlea of P1 *Map3k1^+/+^* (i) and *Map3k1^goya/goya^* (iii) mice. Widespread nuclear expression is seen in both genotypes; however, the depth of staining is greatly increased in *Map3k1^goya/goya^*. Some nuclei in spiral ligament, spiral limbus and basilar membrane in the *Map3k1^+/+^* organ of Corti remained unstained with this length of chromogenic exposure. These results were consistent for littermate controls (*Map3k1^+/+^ n*=2, *Map3k1^goya/goya^ n*=3). (C) Immunoratio was used to quantify the percentage of nuclei stained in each mid turn image. This analysis showed that the *Map3k1^goya/goya^* mice (*n*=3) had significantly increased numbers of positively stained cells when compared to *Map3k1^+/+^* (*n*=2). Bii and iv show increased positive staining for phosphorylated p38 in the spiral ganglion neurons of *Map3k1^goya/goya^* (iv) mice compared to *Map3k1^+/+^* (ii). (D,E) Immunostaining of P1 organ of Corti using anti-phospho JNK and anti-phospho ERK1/2 antibodies, respectively. No differences can be seen between *Map3k1^+/+^* (Di,Ei) or *Map3k1^goya/goya^* (Dii,Eii) mice, although, interestingly, expression of both proteins is mainly observed below the basal surface of inner and outer hair cells, in contrast to the widespread nuclear expression of phosphorylated p38.

Because MAP3K1 has been shown to be involved with activation of all three major MAPK pathways – ERK, JNK and p38 MAPK – we investigated the phosphorylation of these downstream target proteins using the capillary based Simple Western Peggy system (Protein Simple). Protein lysates from P1 inner ears were analysed for both total and phosphorylated ERK1/2, JNK and p38 MAPK. Moreover, given the effects in the eye of *Map3k1* mutations on RB phosphorylation (Mongan et al., 2011), we also assessed total and phospho-RB levels. No differences in phosphorylation were detectable in ERK1/2, JNK or RB. However, a trend towards increased p38 phosphorylation in *Map3k1^goya/goya^* inner-ear lysates was observed (Fig. 7A).

To further investigate p38 MAPK phosphorylation in *Map3k1^goya/goya^* inner ears, we performed immunohistochemistry on sections from P1 mice (Fig. 7B). Anti-phospho-p38 labelling of *Map3k1^goya/goya^* homozygotes showed intense nuclear staining of all cell types in the cochlea duct and SGNs (Fig. 7Biii,Biv). The majority of nuclei in the surrounding structures, such as the spiral ligament and spiral limbus, were also intensely stained. Labelling of *Map3k1^+/+^* mice displayed a similar pattern of nuclear expression; however, under identical experimental conditions, the staining was much weaker and fewer nuclei in the surrounding structures stained positive for phospho-p38 MAPK (Fig. 7Bi,Bii).

We quantified the difference in anti-phospho-p38 staining using the ImageJ plugin, ImmunoRatio, analysing mid-cochlear sections from *Map3k1^goya/goya^* and *Map3k1^+/+^* mice. ImmunoRatio has been designed to diagnostically assess the percentage area of positively DAB-stained nuclei in a given sample; however, the analysis does not take into account intensity of stain. The results show a significant (*P*=0.0126) increase in positive nuclear area in *Map3k1^goya/goya^* mice (*n*=3) when compared with *Map3k1^+/+^* (*n*=2) (Fig. 7C).

We also performed immunohistochemistry with anti-phospho-JNK and anti-phospho-ERK1/2 antibodies (Fig. 7D,E), and, consistent with our Simple Western assay data, no obvious differences in intensity or expression pattern were observed between *Map3k1^goya/goya^* and *Map3k1^+/+^* mice. It is worth noting that these antibodies showed strong labelling beneath the basal surface of the IHCs and OHCs, consistent with the location of SGN neurite extensions at the P1 time point. In addition, low-level anti-phospho-JNK was detected in the cytoplasm and nucleus of cells throughout the organ of Corti of both *Map3k1^goya/goya^* and *Map3k1^+/+^* mice.

## DISCUSSION

We report that an IVS13+2T>C ENU-induced lesion in *Map3k1* is the causative mutation underlying both the EOB and auditory phenotypes in the *goya* mutant. These findings demonstrate a newly identified role for MAP3K1 in auditory function. Similar findings are reported in the parallel study (Yousaf et al., 2015). Mice homozygous for the *goya* mutation and *Map3k1*-null mice each develop supernumerary OHCs, and both show a progressive decline in auditory function resulting in severe hearing loss by 9 weeks of age. The *goya* mutant showed a slower rate of auditory decline, but this is likely due to the different genetic backgrounds of the two mutants, with *goya* crossed to C3H and *tm1Yxia* crossed to C57BL/6J. Apart from the early auditory thresholds, no major differences in phenotype were noted between the *goya* and the *tm1Yxia* alleles. Ultrastructural examination of *Map3k1^goya/goya^* and *Map3k1^tm1Yxia/tm1Yxia^* mutant cochleae uncovered a progressive cellular degeneration in the organ of Corti with an apical-to-basal increase in severity. Cellular loss was first seen in the OHCs, although, by 9 weeks, IHCs and pillar cells were also missing in some mid and mid-basal regions, although not statistically significantly. Mice heterozygous for either the *Map3k1^goya^* or the *Map3k1^tm1Yxia^* allele also developed extra OHCs but, interestingly, they do not show progressive cellular degeneration as seen in the respective homozygotes. Indeed, at 1 year of age, *Map3k1^goya/+^* and wild-type mice had similar auditory thresholds. These data suggest that, within the organ of Corti, MAP3K1 plays multiple roles in cellular development and survival.

We show that MAP3K1 is widely expressed in the inner ear. The expression in OHCs and IHCs, along with Deiters' cells, in the organ of Corti is consistent with the observed phenotype of additional rows of OHCs and OHC degeneration that we observe in *Map3k1* mutants. Our findings of MAP3K1 expression in the cochlea are consistent with the observations of Yousaf et al. (2015) in that they too report expression in Deiters' cells, Reissner's membrane and the stria vascularis. However, we identified some additional sites of expression, including Claudius cells, Hensen cells and Border cells, as well as the basilar membrane. We also observed expression at the apical surface of the vestibular sensory epithelia; however, no overt vestibular dysfunction was detected in either homozygous mutant. It is possible that the normal vestibular function in these mice is a consequence of functional redundancy between MAP3K1 and other MAP3Ks. For example, MAP3K4 is known to activate the same downstream pathways as MAP3K1 (Morrison, 2012). However, a previous study investigating the role of MAP3Ks in testis determination failed to uncover functional redundancy between MAP3K1 and MAP3K4 (Warr et al., 2011).

The *goya* mutation has led to the identification of a new sensorineural deafness locus and it is important to consider *Map3k1* as a candidate gene for both dominant and recessive human deafness loci. Human mutations in *MAP3K1* have been shown to cause 46,XY DSD (Pearlman et al., 2010). Two mutations, including a splice-acceptor mutation and a missense mutation, were identified in two families with 46,XY DSD. Moreover, a further two missense mutations were found in 11 sporadic cases examined. For three of these mutations, MAP3K1 function was studied, including phosphorylation of the downstream targets p38, ERK1/2 and JNK. Two of the mutations increased activation of p38 and ERK, possibly resulting from enhanced binding of RHOA to MAP3K1. However, there are no reports of hearing impairment for any of the individuals carrying MAP3K1 mutations. A possible explanation for the absence of an auditory phenotype in individuals with *Map3k1*-related 46,XY DSD is that they are heterozygous for these mutations; homozygous loss-of-function MAP3K1 mutations in humans might not be viable. We investigated downstream pathways of MAP3K1 in the inner ear of P1 *Map3k1^goya/goya^* mice and observed an increase in p38 phosphorylation, but we did not see any differences in ERK1/2 phosphorylation.

Mice lacking retinoblastoma protein develop extra IHCs and OHCs, and analysis of progenitor cell proliferation indicates that RB1 is involved in cell cycle exit of sensory progenitor cells (Sage et al., 2005). The additional hair cells in *Rb1*-knockout mice can transduce mechanical stimuli, but they undergo apoptosis and are completely missing by 3 months of age (Sage et al., 2006). Similarly, mice lacking p27KIP1, an inhibitor of cyclin-dependent kinases, also develop supernumerary sensory hair cells (Lowenheim et al., 1999). Given the close similarities of the *Map3k1* mutant inner-ear phenotype to that seen in *Rb1* and *p27^kip1^* mutants and the reported effects of a *Map3k1* knockout on *Rb1* signalling in the retina, we surmised that the effects on OHCs observed in both the *Map3k1^goya^* and *Map3k1^tm1Yxia^* mutants are due to a JNK-independent pathway, likely the RB/E2F pathway. However, at the P1 time point investigated, we found no significant changes in the levels of cyclin D1 and cyclin-dependent kinases (CDKs) – the downstream effectors of p27KIP1 – or in phosphorylation levels of RB1 protein. Moreover, we observed no difference in localisation of the anti-proliferative marker p27KIP1 in the developing cochleae of *Map3k1^tm1Yxia/tm1Yxia^* mice and littermate controls. Indeed, an absence of EdU-positive nuclei within the pro-sensory region of *Map3k1^tm1Yxia/tm1Yxia^* cochleae at E14.5 confirms that the ZNP is correctly established in these mice. At E18.5, the continued absence of EdU-positive nuclei within the sensory-cell domain indicates that the p27KIP1-induced cell cycle arrest is maintained in *Map3k1^tm1Yxia/tm1Yxia^* cochleae. These findings suggest that the supernumerary OHCs found in MAP3K1-deficient cochleae do not arise as a consequence of extended or aberrant proliferation of pro-sensory progenitor cells. MAP3K1 is known to act upon a diverse number of molecular pathways, many of which affect cellular proliferation and transcriptional regulation. It is possible that a reduction in MAP3K1 activity leads to dysregulation of genes or proteins involved with cellular fate within the sensory epithelium, or those required for the correct establishment of cell fate boundaries, potentially resulting in additional OHCs. As such, further investigation of the mechanism underlying the auditory phenotype identified in the *goya* mice will require additional transcriptomic and proteomic studies.

In conclusion, we show that, in addition to previously reported eye phenotypes resulting from MAP3K1 deficiency, both heterozygous and homozygous *goya* and *Map3k1*-null mutant mice initially develop supernumerary cochlear OHCs. In homozygous, but not heterozygous, mutants, OHCs progressively degenerate and mice are severely deaf by 9 weeks of age. These phenotypic differences indicate that MAP3K1 might play distinct roles in cochlear development and hair-cell survival. We show increased p38 phosphorylation in the cochleae of *goya* homozygote mice, and EdU studies reveal that the extra OHCs result from a mechanism other than aberrant proliferation. Characterisation of *goya* reveals a signalling molecule that was previously unknown to be involved with mammalian audition, and identifies a candidate gene for human sensorineural hearing loss.

## MATERIALS AND METHODS

### Mice

All animals were housed and maintained under specific pathogen-free (SPF) conditions in individually ventilated cages in the Mary Lyon Centre, MRC Harwell, in adherence to environmental conditions as outlined in the Home Office Code of Practice. Animal procedures were carried out in line with Home Office regulations, and mice were euthanized by Home Office Schedule 1 methods.

The *goya* mutant line was identified from the collaborative ENU mutagenesis vision screen undertaken by MRC MGU Harwell and MRC HGU Edinburgh. ENU-treated G_0_ C57BL/6 male mice were mated to C3H.C-*Pde6b*^+^ female mice to produce G_1_ progeny. G_1_ males were mated to C3H.C-*Pde6b*^+^ female mice to produce G_2_ progeny. Female G_2_ mice were backcrossed to the G_1_ fathers to produce G_3_ mice that were screened for recessively inherited phenotypes. The *goya* line was maintained on a C3H genetic background by outcrossing and intercrossing successive generations. *Map3k1*-null mice (*Map3k1^tm1Yxia/tm1Yxia^*) were imported from Ying Xia's group at the University of Cincinnati College of Medicine (Cincinnati, USA) and rederived by *in vitro* fertilisation by the FESA core in the Mary Lyon Centre to maintain SPF status. The null mice were backcrossed to C57BL/6J.

### Histological analysis

Animals were euthanized and eyes fixed in Davidson's fixative. Fixed specimens were decalcified, dehydrated and embedded in paraffin wax, and 5-µm sagittal sections were obtained and H&E-stained using standard protocols. For inner ears, heads were removed, skinned and bisected along the midline before fixation in 10% neutral buffered formalin (Surgipath), and subsequent processing as above. See Fig. S1.

### Linkage analysis

DNA from the parental strains (C57BL/6J and C3H.C-*Pde6b*^+^) and five affected G3 mice were scanned using an Illumina mouse low-density linkage array employing 271 informative SNPs.

### Mutational analysis of *Map3k1*

Exons and the immediate flanking sequences of *Map3k1* were amplified from *goya*, C57BL/6J and C3H.C-*Pde6b*^+^ genomic DNA employing oligonucleotides that were also used for Sanger sequencing.

### RT-PCR

Total RNA was extracted from microdissected P1 organ of Corti using the RNeasy micro kit (Qiagen). For each sample, RNA was pooled from four ears from two mice. First-strand cDNA was synthesised using a high-capacity cDNA reverse-transcription kit (Life Technologies) using a combination of oligo-(dT), random-hexamer and *Map3k1-*specific primers. The cDNA was then used as a template for PCR amplification using a forward primer spanning the end of exon 11 and the beginning of exon 12 and a reverse primer from exon 14 (primer sequences on request). PCR products were separated by gel electrophoresis, bands excised, cloned into pGEM-T vector, and sequenced using SP6 and T7 primers.

### Gene expression

RNA extractions and cDNA synthesis were performed as described above, except that only random hexamers were used to prime the cDNA synthesis reactions. TaqMan^®^ (Life Technologies) assays for *Ccna1* (cyclin A1), *Ccnd1* (cyclin D1), *Dhfr*, *Map3k1*, *Rb1*, *Col1a1*, *E2f1*, *Fos*, *Jun*, *Axin2*, *CTNNB1* (β-catenin), *Lgr5* and *Gapdh* were run on a 7500 Fast real-time PCR machine (Applied Biosystems) as per the manufacturer's recommended instructions. See Fig. S2.

### Auditory brainstem response

ABR testing was performed as previously described by Hardisty-Hughes et al. (2010). Tone-burst stimuli were presented free-field at 8 kHz, 12 kHz, 20 kHz and 26 kHz to the right ear of the mouse. TDT system III hardware and software (Tucker Davis Technology) was used for stimulus presentation and response averaging, starting at the highest level (90 dB SPL) and reducing in 5 or 10 dB increments until no response trace could be observed. Mice that displayed no response to a 90 dB SPL stimulus were recorded as 100 dB SPL for subsequent analysis.

### Scanning electron microscopy (SEM)

Animals were euthanised and excised inner ears were fixed overnight in 2.5% glutaraldehyde in 0.1 M phosphate buffer (Sigma-Aldrich), then decalcified for 48 h in 4.3% EDTA in 0.1 M phosphate buffer (Sigma-Aldrich). Fine dissection was performed to reveal the organ of Corti, before osmium tetroxide (Agar Scientific)-thiocarbohydrazide (Fluka) (OTOTO) processing (adapted from Hunter-Duvar, 1978) was carried out. Samples were then dehydrated through increasing-strength ethanol solutions (Fisher Scientific) and critical point dried using an Emitech K850 (EM Technologies Ltd). Specimens were then mounted on stubs using silver paint (Agar Scientific) and sputter coated with platinum using a Quorum Q150T sputter coater (Quorum Technologies). Prepared cochleae were visualised with a JEOL LSM-6010 (Jeol Ltd) scanning electron microscope. Hair-cell counts were performed by counting the number of adjacent IHCs and OHCs to 20 pillar cells; for the analysis, the cochlea was divided into four separate regions (turns): apical (<90° from apex), mid-apical (90-180° from apex), mid (180-360° from apex) and mid-basal (360-540° from apex). Ears from at least three mice were analysed for each genotype at each turn and time point.

### X-Gal staining

Mice were euthanized and inner ears removed and fixed for 2 h at 4°C in 0.1 M phosphate buffer containing 1% paraformaldehyde (Sigma-Aldrich), 2 mM MgCl_2_ (Sigma-Aldrich), 0.25% glutaraldehyde (Sigma-Aldrich) and 5 mM EGTA (Merck Millipore). Ears were then washed in 0.1 M phosphate buffer containing 2 mM MgCl_2_ (Sigma-Aldrich) and 0.02% NP-40 (Fluka). Staining was performed overnight at room temperature (RT) in a solution of 0.1 M phosphate buffer containing 2 mM MgCl_2_ (Sigma-Aldrich), 5 mM potassium ferrocyanide (Sigma-Aldrich), 5 mM potassium ferricyanide (Sigma-Aldrich), 0.02% NP-40 and 1 mg/ml 5-bromo-4-chloro-indolyl-β-D-galactopyranoside (X-Gal) (Sigma-Aldrich). Post-staining, ears were decalcified in 4.3% EDTA in 0.1 M phosphate buffer (Sigma-Aldrich) for 48 h at 4°C, before paraffin embedding and sectioning at 10 µm. Sections and whole-mount dissected cochlea were imaged on a Zeiss Axio Observer Z-1 microscope using extended focus image capture.

### Peggy Simple Western size assay

Whole inner ears from three P1 mice (one ear per mouse) were pooled and lysed in 20 mM Bicene with 0.6% Chaps supplemented with phosSTOP™ and cOmplete mini™ inhibitor cocktails (Roche), using a Precellys 24 homogeniser with a soft tissue kit (Precellys).

Capillary-based immunodetection was performed using the automated Peggy™ system (Simple Western™) as described previously (Siggers et al., 2014). Briefly, lysates were mixed with Simple Western™ sample dilution buffer (Protein Simple) containing reducing agent and fluorescent standards, and denatured at 95°C for 5 min. Samples were then loaded into the 384-well assay-plate and proteins were separated through size-resolving matrix, immobilized to the inner capillary wall, incubated with p38 MAPK (CST9212), phospho-p38 MAPK (Thr180/Tyr182) (CST9215), p44/42 MAPK (ERK1/2) (CST9102), phospho-p44/42 MAPK (ERK1/2) (Thr202/Tyr204) (CST4377), SAPK/JNK (CST9252) and phospho-SAPK/JNK (Thr183/Tyr185) (CST9251) primary antibodies and HRP-conjugated secondary antibodies before detection using chemiluminescence.

### Immunohistochemistry

#### Phospho-p38, phospho-JNK and phospho-ERK1/2

P1 mice were euthanized by decapitation and bisected heads fixed in 4% PFA in PBS for 1 h at 4°C. The bisected heads were then dehydrated and embedded in paraffin wax and 5-µm sections collected onto charged slides. Sections were de-paraffinised, endogenous peroxidase activity quenched by submersion in 3% H_2_O_2_, washed in 1× TBST and blocked in 1× TBST containing normal goat serum. Sections were then incubated overnight at 4°C with rabbit monoclonal anti-phospho p38 MAPK (Thr180/Tyr182) (Cell Signaling Technology) at 1:1500 dilution, phospho-p44/42 MAPK (ERK1/2) (Cell Signaling Technology) at 1:1000 dilution or phospho-SAPK/JNK (Thr183/Tyr185) (Cell Signaling Technology) at 1:1000 dilution. The VECTASTAIN^®^ Elite ABC rabbit IgG avidin biotin kit (Vector Laboratories) and DAB+ Chromagen (Dako) were used for detection. For anti-phospho-p38-abelled images, the ImmunoRatio plugin (http://jvsmicroscope.uta.fi/immunoratio/) for ImageJ (http://imagej.nih.gov/ij/) was used to quantify the percentage of positively stained nuclei.

#### Proliferation detection and p27KIP1 immunofluorescence

The Click-iT Plus™ EdU Alexa-Fluor-594 Imaging Kit (Life Technologies) was used to identify proliferating cells in embryonic cochlea. Pregnant females were injected with 100 µg 5-ethynyl-2′-deoxyuridine (EdU) in 200 µl PBS twice, at 2 h intervals, before embryos were harvested 2 h after final injection at either E14.5 or E18.5. Embryonic heads were fixed in 4% PFA in PBS for 1 h at 4°C and tail collected for genotyping. Fixed heads were then dehydrated and embedded in paraffin wax and 5-µm sections collected onto charged slides. The copper-azide ‘click’ Alexa-Fluor-594 reaction for detection of EdU was performed as per the manufacturer's instructions, and processed slides were washed in PBS containing 3% BSA at RT. Slides were then blocked with 1× PBS containing 5% donkey serum and 0.5% Triton X-100 (Sigma), before incubation with rabbit polyclonal anti-p27KIP1 (Cell Signaling Technology) at 1:200 dilution overnight at 4°C. Slides were washed in PBS before incubation with Alexa-Fluor-488-conjugated donkey anti-rabbit secondary antibody at 1:200 dilution. Fluorescent confocal images were collected using a Zeiss LSM 700 inverted microscope.

### Statistical analysis

#### Within-genotype rates of hair-cell loss across weeks 2, 4 and 9

Counts were split into six distinct datasets for model fitting, according to hair-cell type (inner or outer) and genotypic group. A Poisson generalised linear model was fitted to each dataset separately. The model was specified as:

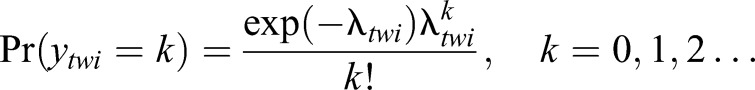


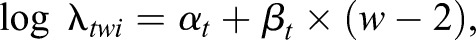


(i.e. with a turn-specific, log-linear relationship between mean hair-cell count and week), where

- *t*∈{1, 2, 3, 4} indexes turn (1=apical, 2=mid-apical, 3=mid, 4=mid-basal),
- *w*∈{2, 4, 9} denotes week,
- *i*∈{1, 2,…, *n_tw_*} indexes mouse within (turn, week) group,
- *y_twi_* is the observed hair-cell count in week *w*, at turn *t*, in mouse *i*, and
- *λ_twi_* is the mean hair-cell count in week *w*, at turn *t*, in mouse *i*.

For estimates and confidence intervals for the weekly percentage change in mean hair-cell count at each turn *t* {i.e. 100×[exp(*β_t_*)−1]}, see Table S1. Table S2 displays *P*-values from testing the null hypothesis that the weekly percentage change is zero (i.e. *H*_0_:*β_t_*=0). Application of a variety of diagnostic tools suggested that the model provided a reasonably good fit to the data.

Model fitting, diagnostic plots and hypothesis tests were performed using the glm()-based functionality of the package ‘stats’ in R (R Core Team, 2013).

#### Inter-genotype comparison of hair-cell counts at week 2

For each cell type (inner and outer), and for each turn, cell counts were compared pair-wise between genotypic groups; *P*-values result from a Welch *t*-test applied to log-transformed hair-cell counts.

#### Comparison of qualitative phenotypes across genotypic groups

The proportion of mice carrying each particular qualitative phenotype (Normal, Extra row or Extra OHC) was estimated in each genotypic group (*Map3k1^+/+^*, *Map3k1^goya/+^* and *Map3k1^tm1Yxia/+^*). Phenotype proportions were compared pair-wise across genotypes to determine whether mice with a particular phenotype were over-represented in some genotypic groups relative to others. Specifically, estimates and exact binomial confidence intervals were obtained for the proportion of mice of a particular genotype carrying a particular phenotype (Brown et al., 2001). Fisher's exact test was used to test the null hypothesis of equality of phenotypic proportion across a pair of genotypic groups (for estimates, confidence intervals and *P*-values, see Table S3).
